# Galangin Reduces the Loss of Dopaminergic Neurons in an LPS-Evoked Model of Parkinson’s Disease in Rats

**DOI:** 10.3390/ijms19010012

**Published:** 2017-12-21

**Authors:** Guangxin Chen, Juxiong Liu, Liqiang Jiang, Xin Ran, Dewei He, Yuhang Li, Bingxu Huang, Wei Wang, Shoupeng Fu

**Affiliations:** College of Animal Science and Veterinary Medicine, Jilin University, Changchun 130062, China; chengx15@mails.jlu.edu.cn (G.C.); juxiong@jlu.edu.cn (J.L.); jianglq9914@mails.jlu.edu.cn (L.J.); ranxin9914@mails.jlu.edu.cn (X.R.); hedw9914@mails.jlu.edu.cn (D.H.); yhli9915@mails.jlu.edu.cn (Y.L.); huangbingxu16@mails.jlu.edu.cn (B.H.); wangwei@jlu.edu.cn (W.W.)

**Keywords:** Parkinson’s disease, galangin, microglia, MAPKs, NF-κB, AKT

## Abstract

Parkinson’s disease (PD) is caused by the loss of dopaminergic (DA) neurons in the midbrain substantia nigra (SN). Neuroinflammation, which is marked by microglial activation, plays a very important role in the pathogenesis of PD. Pro-inflammatory mediators produced by activated microglia could damage DA neurons. Hence, the inhibition of microglial activation may provide a new approach for treating PD. Galangin has been shown to inhibit inflammation in a variety of diseases, but not PD. In this study, we aimed to investigate the anti-inflammatory effect of galangin and the underlying mechanisms in Lipopolysaccharide (LPS) induced PD models. We first examined the protective effect of galangin in the LPS-induced PD rat model. Specifically, we investigated the effects on motor dysfunction, microglial activation, and the loss of DA neurons. Then, galangin was used to detect the impact on the inflammatory responses and inflammatory signaling pathways in LPS-induced BV-2 cells. The in vivo results showed that galangin dose-dependently attenuates the activation of microglia, the loss of DA neurons, and motor dysfunction. In vitro, galangin markedly inhibited LPS-induced expression of tumor necrosis factor α (TNF-α), interleukin-6 (IL-6) and interleukin-1β (IL-1β), cyclooxygenase 2 (COX-2), and induced nitric oxide synthase (iNOS) via associating with the phosphorylation of c-JUN N-terminal Kinase (JNK), p38, protein kinase B (AKT), and nuclear factor κB (NF-κB) p65. Collectively, the results indicated that galangin has a role in protecting DA neurons by inhibiting microglial activation.

## 1. Introduction

Parkinson’s disease (PD) is one of the common neurodegenerative disorders and affects over 4 million people worldwide [1]. The characteristic feature of PD is the loss of DA neurons in the midbrain substantia nigra (SN), which leads to bradykinesia, postural instability, resting tremor, and rigidity of patients [2,3,4]. To date, the pathogenesis of PD is still unknown. Although existing treatments provide benefits for PD patients, medications to cure or slow the progress of PD are still poor. Neuroinflammation was reported to be an important mechanism responsible for the pathogenesis of PD [5,6]. Normal neuroinflammation is important to protect the central nervous system (CNS). However, uncontrolled and prolonged neuroinflammation is potentially harmful and can cause damage of DA neurons [7]. The mark of neuroinflammation is the activation of microglia. Pro-inflammatory mediators, produced by activated microglia, are responsible for the damage to DA neurons. Hence, inhibition of over-activation of microglia may provide a new approach for treating PD. 

Galangin is a member of the flavonol subclass of flavonoids, which are present at high concentrations in the rhizome of *Alpinia officinarum* and have been applied for traditional Chinese medicine for various diseases [8,9]. Galangin has been reported to have certain biological activities, including antibacterial [10], anticancer [11], anti-obesity [12], and antioxidant activities [13,14]. Previous studies also indicated that galangin suppresses LPS-induced inflammation in macrophages [15] and inhibited inflammation in cisplatin-induced nephrotoxicity [14], collagen-induced arthritis [16], mast cell-mediated allergic inflammation [17], LPS-induced acute lung injury [18], and ovalbumin-induced airway inflammation [19]. Since galangin suppresses inflammation in various diseases, it may have an anti-neuroinflammatory effect in PD. However, there are no reports supporting this idea. Therefore, the aim of this study was to evaluate the neuroprotective and anti-inflammatory effect of galangin in LPS-induced PD models and to reveal the anti-inflammatory mechanism of galangin.

## 2. Results

### 2.1. Galangin Inhibited the Activation of Microglial Cells in an LPS-Induced PD Rat Model

**Figure 1:**
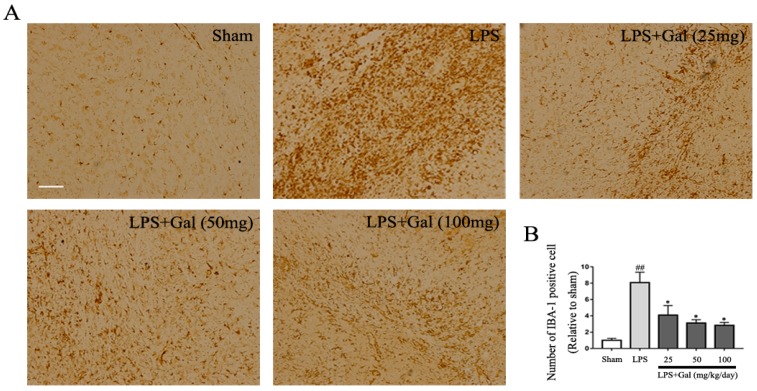

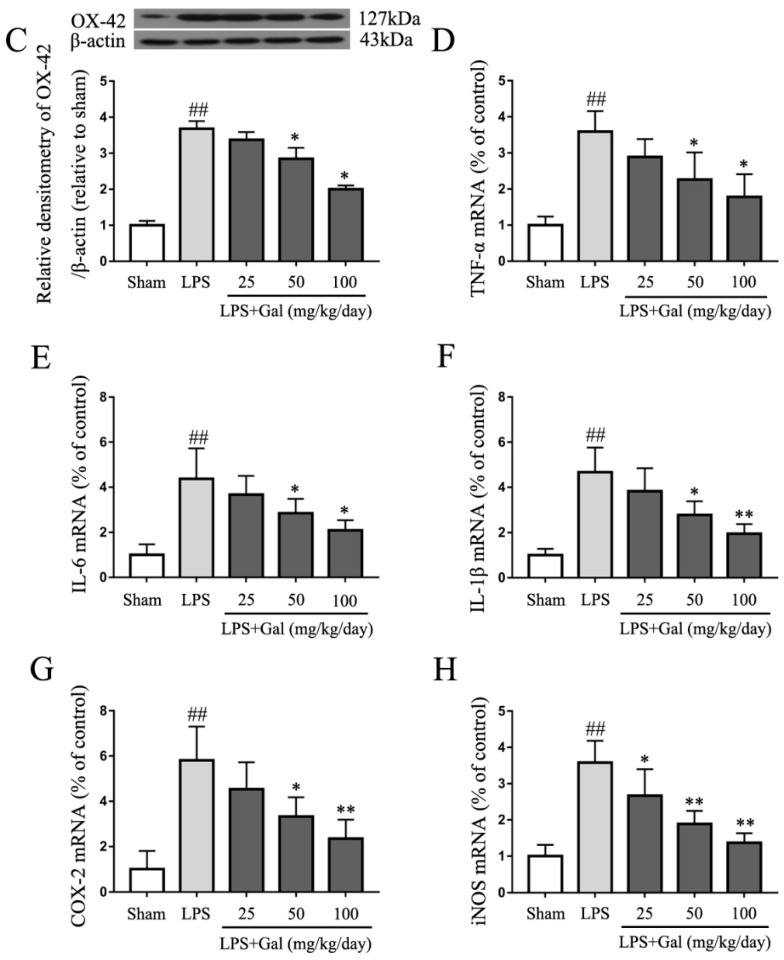
Galangin inhibited the activation of microglial cells in an LPS-induced Parkinson’s disease (PD) rat model. All rats were randomly divided into five groups and each of the groups contained 12 rats: the sham-operated group; LPS treatment group, the right substantia nigra pars compacta (SNpc) received 10 μg LPS at the concentration of 5 μg/μL; LPS and galangin (25, 50, and 100 mg/kg/day) treatment group, galangin was dissolved in sterile saline containing 5% Tween 80 (*v*/*v*) and was administered through gavage beginning 3 days before LPS injection and for 28 days in total. (**A**) Immunohistochemistry analysis of the microglial cells in SN with IBA-1 antibody, magnification shown is 10×, the scale bar represents 300 μm (*n* = 4 per group); (**B**) The number of IBA-1 positive cells (*n* = 4 per group); (**C**) The expression of OX-42 was analyzed by western blot (*n* = 4 per group); (**D**–**H**) The mRNA expression of *TNF-α*, *IL-6*, *IL-1β*, *COX-2*, and *iNOS* (*n* = 4 per group). Data are show as means ± SD. ## *p* < 0.01 compared to the sham group, * *p* < 0.05 and ** *p* < 0.01 compared to the LPS group.

Previous studies have shown that galangin has anti-inflammatory functions in various diseases. Therefore, in this investigation, we first measured the effect of galangin on microglial activation in an LPS-induced PD rat model. Immunohistochemistry results showed that LPS dramatically increased the number of IBA-1 positive cells, and galangin could dose-dependently decrease the number of IBA-1 positive cells (Figure 1A,B). The results indicated that galangin dose-dependently inhibited the activation of microglial cells in an LPS-induced rat model. To further prove the results, we measured OX-42, another marker of microglial activation, by western blot. The western blot results showed that LPS markedly increased the expression of cluster of differentiation 11b (CD11b, OX-42), and galangin significantly decreased its expression in a dose-dependent manner (Figure 1C). Next, we measured the expression of pro-inflammatory mediators in the SN of an LPS-induced PD rat model. Galangin dramatically decreased the expression of *TNF-α*, *IL-6*, *IL-1β*, *COX-2*, and *iNOS* induced by Lipopolysaccharide (LPS) (Figure 1D–H). Collectively, this data indicated that galangin inhibited the activation of microglial cells in an LPS-induced PD rat model.

### 2.2. Galangin Suppresses the Loss of DA Neurons in an LPS-Induced PD Rat Model

**Figure 2:**
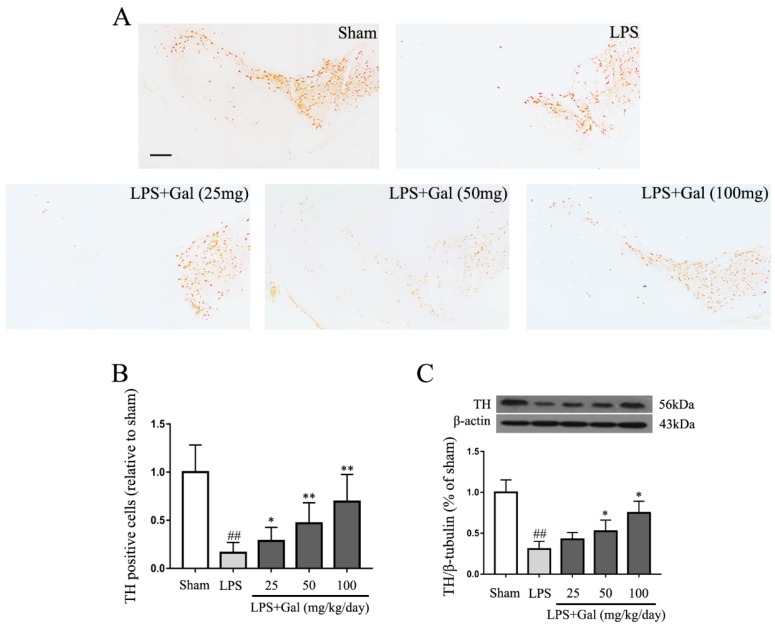
Galangin suppresses the loss of DA neurons in an LPS-induced PD rat model. The treatment is identical to what is described in Figure 1. (**A**) Staining of tyrosine hydroxylase (TH)-positive neurons in the SN with immunohistochemistry analysis, magnification shown is 4×, the scale bar represents 100 μm (*n* = 4 per group); (**B**) The survival ratio of the dopaminergic neurons in the SNpc (TH positive cells) (*n* = 4 per group); (**C**) The expression of TH was analyzed by western blot (*n* = 4 per group). Data are means ± SD. ## *p* < 0.01 compared to the sham group, * *p* < 0.05 and ** *p* < 0.01 compared to the LPS group.

Many reports have shown that PD is accompanied by the loss of DA neurons. We have proven that galangin inhibited the activation of microglial cells in an LPS-induced rat model. To assess whether galangin protects DA neurons via its anti-inflammatory activity, we measured the expression of tyrosine hydroxylase (TH) by immunohistochemical staining. The experimental results showed that the number of TH-positive neurons dramatically decreased in the LPS treated group, while galangin dose-dependently attenuated LPS-induced loss of TH-positive neurons (Figure 2A,B). The western blot results also showed that galangin attenuated the LPS-induced decline of TH in an LPS-induced PD rat model (Figure 2C). 

### 2.3. Galangin Attenuated LPS-Induced Motor Dysfunction in an LPS-Induced PD Rat Model

**Figure 3:**
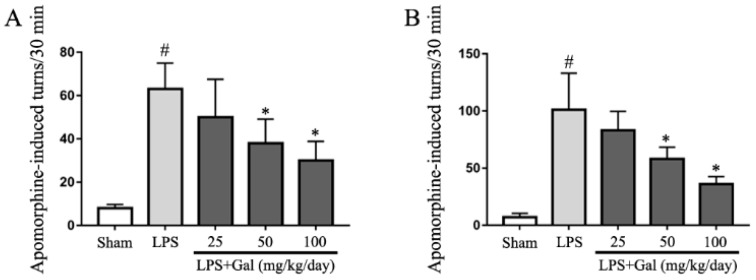
Galangin attenuated LPS-induced motor dysfunction in rat. (**A**,**B**) The number of turns at 14 days (**A**) and 28 days (**B**) was induced by apomorphine in an LPS-induced PD rat model (*n* = 12 per group). Data are shown as means ± SD. # *p* < 0.05 compared to the sham group, * *p* < 0.05 compared to the LPS group.

To further investigate whether galangin treatment has a neuroprotective role in an LPS-induced PD rat model, the rotational behavior assay was performed. After galangin was administered via gavage for 3 days, LPS was injected into the right SN of the rats. To detect the effect of galangin on motor dysfunction, LPS-induced PD rats were subjected to rotational behavior at 14 and 28 days. Animals underwent intraperitoneal injection of amphetamine, and beginning 5 min after injection, the number of turns within a 30 min period was recorded. The results showed that galangin dose-dependently decreased amphetamine-induced turns in an LPS-induced PD rat model (Figure 3A,B).

### 2.4. Galangin Suppresses the LPS-Induced Inflammatory Response in BV-2 Cells

**Figure 4:**
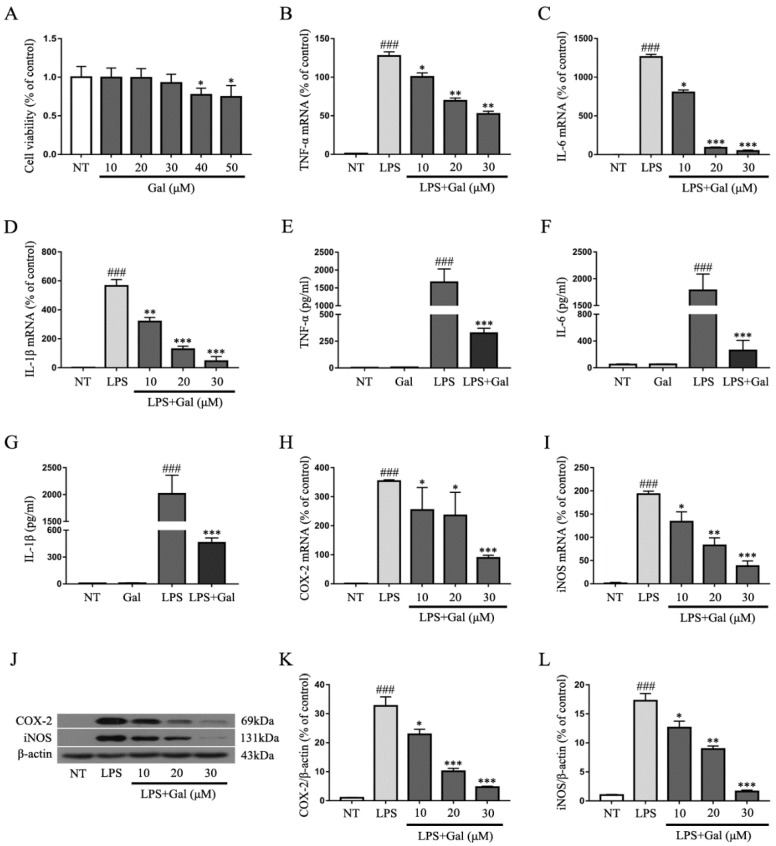
Galangin suppresses the LPS-induced inflammatory response in BV-2 cells. (**A**) The effects of galangin on BV-2 cell viability (*n* = 5); (**B**–**D**,**H**,**I**) The BV-2 cells were stimulated with LPS for 4 h after pretreating with galangin for 1 h, and then cells were collected with Trizol. Gene expression of *TNF-α*, *IL-6*, *IL-1β*, *COX-2*, and *iNOS* was detected by qRT-PCR (*n* = 3). The BV-2 cells were stimulated with LPS for 12 h after pretreating with galangin for 1 h, and then, the supernatant was collected. The protein levels of TNF-α, IL-6 and IL-1β were analyzed by ELISA (**E**–**G**) (*n* = 3); the protein levels of COX-2 and iNOS were analyzed by western blot (**J**–**L**) (*n* = 3). Data are shown as means ± SD. ### *p* < 0.001 compared to the no-treatment group (NT), * *p* < 0.05, ** *p* < 0.01, and *** *p* < 0.001 compared to the LPS group.

Our study indicated that galangin protected DA neurons by inhibiting microglial activation in an LPS-induced PD rat model. Next, we measured the anti-inflammatory effect of galangin in LPS-induced BV-2 cells. A cell viability assay showed that 10, 20, and 30 μg/mL of galangin had no effects on cell viability, but 40 and 50 μg/mL of galangin decreased the cell viability (Figure 4A). Therefore, 30 μg/mL of galangin was thought to be the highest concentration without affecting the cell viability. BV-2 cells were stimulated with LPS (1 μg/mL) for 4 h after pretreating with various concentrations of galangin for 1 h. Cells were collected with Trizol. The mRNA levels of pro-inflammatory mediators were measured by qRT-PCR. The qRT-PCR results showed that galangin decreased the LPS-induced gene expression of pro-inflammatory mediators *TNF-α*, *IL-6*, *IL-1β*, *COX-2*, and *iNOS* (Figure 4B–D,H,I). Furthermore, cells were stimulated with LPS for 12 h after pretreating BV-2 cells with galangin for 1 h. The supernatant was collected. The protein levels of pro-inflammatory mediators were detected by ELISA or western blot. The ELISA results showed that galangin inhibited the release of the pro-inflammatory cytokines TNF-α, IL-6 and IL-1β in LPS-induced BV-2 cells (Figure 4E–G). The western blot results showed that galangin significantly decreased the protein levels of pro-inflammatory enzymes COX-2 and iNOS in a dose-dependent manner (Figure 4J–L).

### 2.5. Galangin Associated with the Phosphorylation of the NF-κB p65, AKT, and MAPKs Signaling Pathways in LPS-Induced BV-2 Cells

**Figure 5:**
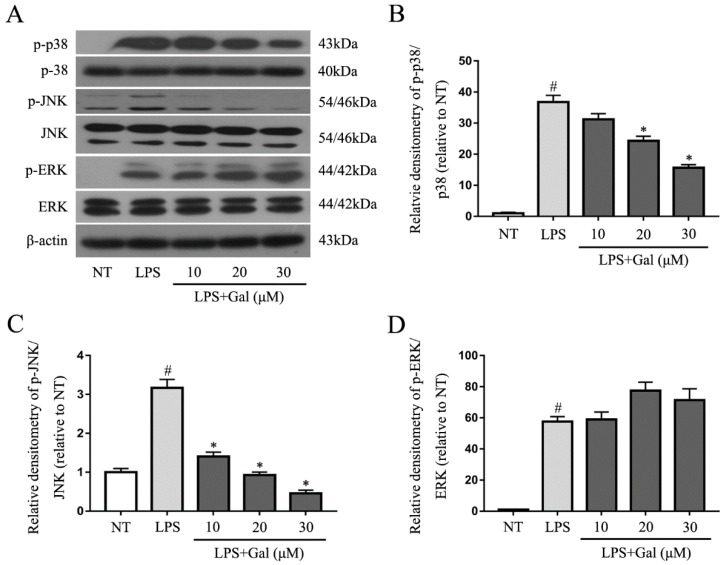
Galangin inhibited the phosphorylation of p38 and JNK. (**A**) Western blot analysis for the phosphorylation of p38, JNK, and ERK (*n* = 3). Cells were pretreated with various concentrations of galangin (10, 20, and 30 μM) for 1 h, followed by stimulation with LPS for 1 h, then collected. Western blot analysis was performed using antibodies against phospho- or total forms of ERK, p38, and JNK (**B**–**D**) Quantification of western blot data of p-p38, p-JNK, and p-ERK. Data are shown as means ± SD. # *p* < 0.05 compared to the NT group, * *p* < 0.05 compared to the LPS group.

**Figure 6:**
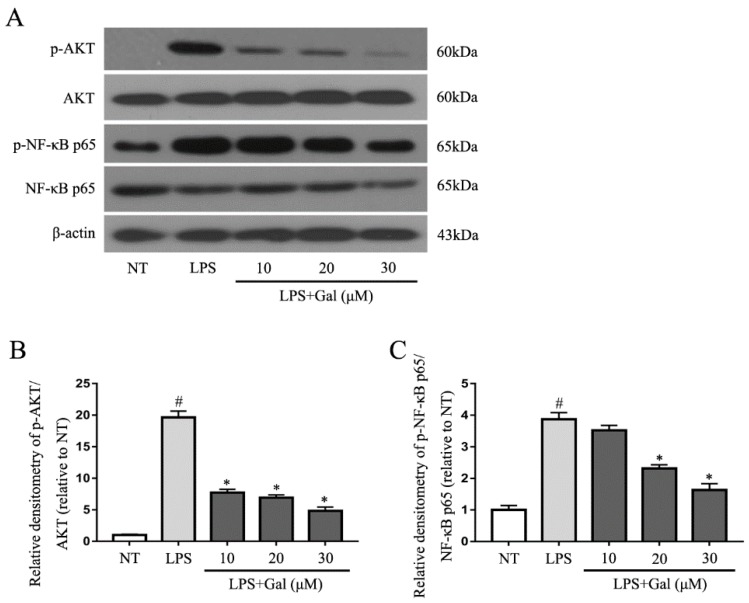
Galangin inhibited the phosphorylation of NF-κB p65 and AKT. (**A**) Western blot analysis for the phosphorylation of NF-κB p65 and AKT (*n* = 3). Cells were pretreated with various concentrations of galangin (10, 20, and 30 μM) for 1 h, followed by stimulation with LPS for 1 h, then collected. Western blot analysis was performed using antibodies against phospho- or total forms of AKT and NF-κB p65; (**B**,**C**) The relative densitometry of p-NF-κB p65 and p-AKT (relative to normal). Data are shown as means ± SD. # *p* < 0.05 compared to NT, * *p* < 0.05 compared to the LPS group.

Mitogen-activated protein kinases (MAPKs) and NF-κB signaling pathways are two classical inflammation signaling pathways. Inhibition of the phosphorylation of these signaling pathways would suppress the inflammatory response in LPS-induced BV-2 cells. In our study, we first examined the MAPKs signaling pathway and found that galangin dramatically associated with LPS-induced phosphorylation of JNK and p38, but not ERK (Figure 5). We then measured NF-κB and its upstream signaling pathway, AKT. Galangin also significantly associated with the phosphorylation of p65 and AKT (Figure 6).

## 3. Discussion

Our present study showed that galangin inhibits the loss of DA neurons via reducing the activation of microglia in an LPS-induced PD rat model. Further study found that galangin significantly inhibits the production of TNF-α, IL-6, IL-1β, COX-2, and iNOS in LPS-induced BV-2 cells. The mechanistic studies found that galangin suppresses the activation of microglial cells via associating with phosphorylation of the AKT, NF-κB p65, JNK, and p-38 signaling pathways.

Galangin is one of the flavonoids that is found in *Alpinia officinarum* and is used as a form of herbal medicine for a variety of ailments [8,9]. Galangin had been used for its antibacterial, anticancer, anti-obesity, and antioxidant activities [10,11,13,14,15]. Previous studies also indicated that galangin suppresses inflammation in LPS-induced macrophages via inhibiting phosphorylation of ERK [15]. Galangin ameliorates cisplatin-induced nephrotoxicity by attenuating oxidative stress, inflammation, and cell death through inhibition of the ERK and NF-κB signaling pathways [14]. Galangin inhibits osteoclastic bone destruction and osteoclastogenesis by inhibiting the phosphorylation of NF-κB in collagen-induced arthritis and bone marrow-derived macrophages [16]. Galangin dampens LPS-induced acute lung injury via inhibiting NF-κB and upregulating heme oxygenase (HO)-1 [18]. These studies demonstrated that galangin has an important role in inhibiting various forms of inflammation. Neuroinflammation is one of the most important causes of PD. Hence, galangin is a potential anti-neuroinflammatory drug that could prevent or slow the progression of PD, and this study proved that.

In rodent experiments, peripheral challenge with LPS activates microglial cells, which are the major active immune cells in the CNS [20]. The activation of microglia cells can produce NO, PGE2, TNF-α, IL-1β, and IL-6, which can contribute to the inflammatory reaction in neurodegenerative diseases [21,22]. The LPS-induced PD rat model, which injects LPS unilaterally into the substantia nigra pars compacta (SNpc), is widely used to study the inflammatory process in the pathogenesis of PD [23,24,25]. Therefore, to elucidate whether galangin has anti-inflammation effects in an LPS-induced PD rat model, we first measured IBA-1 and OX-42, which are biomarkers of microglial activation [26]. The immunohistochemical staining results showed that LPS dramatically increased the activation of microglia, and this effect is abolished by galangin in a dose-dependent manner. The western blot results also showed that galangin dose-dependently suppressed the expression of OX-42 induced by LPS. These data indicated that galangin inhibited LPS-induced activation of microglia. We then measured the expression of TH which is a rate-limiting enzyme broadly expressed in DA neurons [27] and is used for the synthesis of catecholamines that are critical for the synthesis of dopamine [28]. The measured TH in the SN reflected the extent of the lesions to DA neurons. In this study, we found that galangin dose-dependently increased the number of TH-positive neurons and the protein level of TH in an LPS-induced PD rat model. These data indicated that galangin suppressed the loss of DA neurons induced by LPS. When injected into the right SN of rats, LPS activated the microglial cells, leading to the loss of DA neurons. The unilateral loss of DA neurons caused the compensatory hyperactivity of dopamine receptors, leading to an unbalanced receptor activity. Activation of the receptors with agonist resulted in a rotation behavior towards the injected side. Amphetamine, as an indirect agonist of dopamine receptors, induced rotational behavior in an LPS-induced PD rat model which involves unilateral loss of DA neurons [29,30]. Our study indicated that galangin inhibited the loss of DA neurons in an LPS-induced PD rat model. We then measured the effects of galangin in amphetamine-induced rotation behavior in an LPS-induced PD rat model. The results showed that galangin dose-dependently decreased amphetamine-induced rotational behavior in an LPS-induced PD rat model. Collectively, these data indicated that galangin inhibited the loss of DA neurons via downregulating the activation of microglia.

**Figure 7:**
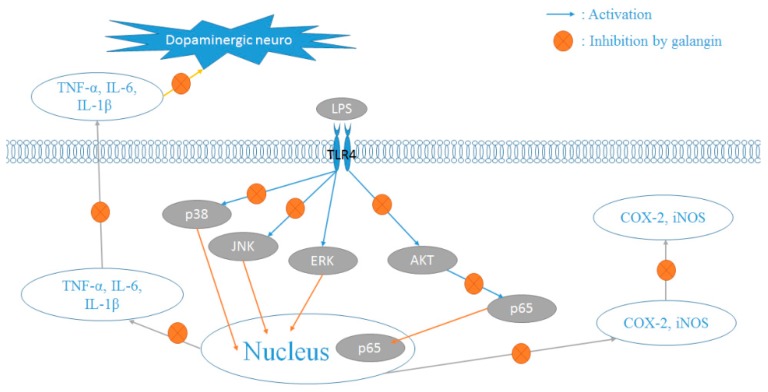
Galangin suppresses pro-inflammatory mediators in LPS-induced BV-2 cells via inhibiting the phosphorylation of the NF-κB p65, AKT, p38, and JNK signaling pathways.

BV-2 cells have a high similarity with microglia cells and share many of the same biomarkers [31]. These cells are the most commonly used alternative to primary microglia cells [32]. In this study, BV-2 cells were used to replace primary microglia cells to investigate the anti-inflammatory mechanisms of galangin. The results indicated that galangin dramatically decreased LPS-induced production of TNF-α, IL-6, IL-1β, COX-2, and iNOS. The results indicated galangin inhibited the inflammation in LPS-induced BV-2 cells. However, the underlying mechanism of galangin suppressed neuroinflammation is still unknown. Hence, to further study the mechanism of galangin inhibited LPS-induced inflammatory response in microglia, we examined the MAPKs and NF-κB signaling pathways. These pathways are crucial for generating an inflammatory response [33], and they modulate LPS-induced expression of IL-1β, IL-6, TNF-α, COX-2, and iNOS in microglia [34]. The inhibition of MAPK and NF-κB signaling is able to suppress the expression of pro-inflammatory mediators in LPS-induced BV-2 cells [24]. In our study, we found that galangin dramatically associated with LPS-induced phosphorylation of p38, JNK, p65, and AKT. Therefore, this study suggests that galangin suppresses the inflammation in an LPS-induced PD model via associating with NF-κB p65, AKT, p38, and JNK, but not ERK (Figure 7).

## 4. Material and Methods

### 4.1. Reagent

Galangin, a yellow powder with 99.4% purity, was purchased from Shanghai Yuanye Bio-Technology Co., Ltd. (Shanghai, China).

### 4.2. Animals and Treatment

Wistar rats that weighted approximately 250 g were provided by the Center of Experimental Animals of the Baiqiuen Medical College of Jilin University (Changchun, China). Animals were maintained under specific pathogen-free conditions. The rats were maintained at 22–24 °C with a 12-h dark/light cycle and were provided with food and water ad libitum. Rats were randomly divided into five groups and each group contained 12 rats: in the LPS treatment group, LPS (5 μg/μL) was dissolved in phosphate buffered saline (PBS) and 2 μL was injected into the right SNpc with the aid of a stereotaxic apparatus; in the LPS and galangin (25, 50, and 100 mg/kg) treatment groups, galangin was dissolved in sterile saline containing 5% Tween 80 (*v*/*v*) and administrated via gavage at various dosages once a day beginning 3 days prior to LPS injection and for 28 days in total; in the sham-operated group, the rats were subjected to the same surgical procedures, except that 2 μL PBS was injected into the right SNpc. Studies were performed in accordance with the guidelines established by the Jilin University Institutional Animal Care and Use Committee (Protocol No. 2015047, 27 February 2015 ).

### 4.3. Rotational Behaviour Assay

To assess lesion severity, the rats were intraperitoneally injected with apomorphine, and rotational behavior was detected at 14 and 28 days, as previously described [25,35]. Briefly, rats were placed in a circular test arena and allowed to adapt to the testing environment for a short period; the animals were then injected with the dopamine receptor agonist apomorphine. The number of turns was noted 5 min after apomorphine was administered and recorded for the total 30 min testing period.

### 4.4. Immunohistochemistry Analysis

Immunohistochemistry was performed as described below. The midbrains were fixed in 4% formaldehyde, embedded in paraffin and sectioned (3 μm per slice). After deparaffinization with xylol, the slices were hydrated with various concentrations of ethyl alcohol. Antigen retrieval was performed at 95 °C in citrate buffer for 5 min. After the citrate buffer cooled to room temperature, the slides were washed three times with PBS. The immunohistochemistry process was performed with an Ultra-Sensitive^TM^ S-P kit (contains endogenous peroxidase blocking solution, sheep serum, biotin-labelled goat anti-rat secondary antibody, and streptavidin-peroxidase) (MBX Biotechnologies, Fuzhou, China). Slides were incubated with endogenous peroxidase blocking solution for 10 min and were washed three times with PBS. This washing was followed by incubation with sheep serum for 1 h. Slides were incubated with anti-TH (1:1000; Abcam, Cambridge, CA, USA) and IBA-1 (1:1000, Proteintech, Chicago, IL, USA) at 4 °C overnight. The slides were successively washed three times with PBS, followed by incubation with biotin-labelled goat anti-rat secondary antibody for 10 min, and washed three times with PBS. This washing was followed by incubation with streptavidin-peroxidase for 10 min, and then the slides were washed three times with PBS. The immune-reactivity was detected using a DAB kit (MBX Biotechnologies, Fuzhou, China). TH and IBA-1 positive cells in the SN were counted by three researchers, and the averages were reported.

### 4.5. Western Blot Analysis

Complete Dulbecco’s Modified Eagle’s Medium (DMEM) (Gibco, Grand Island, NY, USA) was replaced with serum-free DMEM medium to starve the BV-2 cells for 4 h. Cells were pretreated with various concentrations of galangin for 1 h, followed by treatment with LPS (1 μg/mL) for different times. After the last behavioral test, the SNs of the rats were rapidly dissected out, frozen, and stored in a deep freezer at −80 °C until the assays were performed. The BV-2 cells and the rat SNs were lysed with lysis buffer for 30 min (Beyotime Inst. Biotech, Beijing, China). After centrifugation at 12,000× *g* at 4 °C, the supernatant protein was quantified with a bicinchoninic acid protein assay kit (Beyotime Inst. Biotech, Beijing, China). The detailed process is referenced in our previous work [24]. The source of the commercially available antibodies used for the western blot were OX-42 (1:1000), TH (1:1000), COX-2 (1:1000), iNOS (1:2000) (Abcam, Cambridge, CA, USA), phosphor-NF-κB p65 (1:1000), NF-κB p65 (1:1000), phosphor-AKT (1:2000), AKT (1:2000), phosphor-p38 (1:2000), p38 (1:1000), phosphor-ERK1/2 (1:2000), ERK1/2 (1:2000), phosphor-JNK1/2 (1:1000), JNK1/2 (1:2000), (Cell Signaling Technology, Danvers, MA, USA), and β-tubulin (1:2000) (Santa Cruz Biotechnology Inc., Santa Cruz, CA, USA). 

### 4.6. Cell Viability Assay

The microglial cell line (BV-2) was purchased from the Cell Bank of Chinese Academy of Sciences (Shanghai, China). The cells were cultured in complete Dulbecco’s modified Eagle’s medium (DMEM), consisting of 10% (*v*/*v*) fetal bovine serum (FBS) (Clark, Claymont, DE, USA), and incubated at 37 °C in a humidified atmosphere of 5% CO_2_. 3-(4,5-dimethyl-2-thiazolyl)-2,5-diphenyl-2-H-tetrazolium bromide (MTT) assay was used to determine the effects of galangin on the viability of BV-2 cells. Cells were seeded at a density of 1 × 10^4^ cells/well in a 96-well plate and incubated overnight. The medium was replaced with new complete medium, and various concentrations of galangin were added for 24 h. MTT (5 mg/mL) was added to each well, then incubated for 4 h. Dimethyl sulfoxide (DMSO) (Sigma-Aldrich, St. Louis, MO, USA) was added (100 μL) to each well to dissolve the crystals and measured at 570 nm. Five replicates were carried out for various concentrations of galangin.

### 4.7. Quantitative Real-Time PCR

The detailed process of qRT-PCR is referenced in our previous work [36]. Briefly, total RNA was extracted from the BV-2 cells using Trizol (Invitrogen, Carlsbad, CA, USA) according to the supplier’s protocol, and the cDNA was made by a commercial RT-PCR Kit (Takara Shuzo Co., Ltd., Kyoto, Japan). The real-time PCR was then analyzed with SYBR Green QuantiTect RT-PCR Kit (Roche, South San Francisco, CA, USA), and each of the samples was analyzed in triplicate. The sequence of primers used in this investigation is shown in Table 1 and Table 2.

### 4.8. ELISA

The BV-2 cells were seeded in 24-well plate and cultured overnight. The cells were starved with serum-free medium for 4 h. The cells were pretreated with various concentrations of galangin for 1 h, then the cells were treated with LPS (1 μg/mL) for another 12 h. Subsequently, the medium was collected and centrifuged at 12,000 × *g* for 10 min. The supernatant was used to measure the levels of TNF-α, IL-6, and IL-1β by BioLegend ELISA kit (San Diego, CA, USA) according to the manufacturer’s instructions.

### 4.9. Statistics

The results are presented as mean ± SD. All western blot results were analyzed with Image J (National Institutes of Health, Bethesda, MD, USA). Data were analyzed using the statistical software package SPSS 12.0 (SPSS Inc., Chicago, IL, USA). Groups were compared by one-way analysis of variance (ANOVA) followed by the least significant difference test. All bar charts were performed with GraphPad Prism (v 5; GraphPad Software, Inc., La Jolla, CA, USA). #, * *p* < 0.05 was considered significant, and ##, ** *p* < 0.01 was considered markedly significant.

## Figures and Tables

**Table 1:** The primer sequences of *TNF-α*, *IL-1β*, *IL-6*, *iNOS*, *COX-2*, and *β-actin* (mice).

Gene	Sequence	Length (bp)
*TNF-α*	(F): 5′-GCAACTGCTGCACGAAATC-3′ (R): 5′-CTGCTTGTCCTCTGCCCAC-3′	136
*IL-1β*	(F): 5′-GTTCCCATTAGACAACTGCACTACAG-3′ (R): 5′-GTCGTTGCTTGGTTCTCCTTGTA-3′	139
*IL-6*	(F): 5′-CCAGAAACCGCTATGAAGTTCC-3′ (R): 5′-GTTGGGAGTGGTATCCTCTGTGA-3′	138
*iNOS*	(F): 5′-GAACTGTAGCACAGCACAGGAAAT-3′ (R): 5′-CGTACCGGATGAGCTGTGAAT-3′	158
*COX-2*	(F): 5′-CAGTTTATGTTGTCTGTCCAGAGTTTC-3′ (R): 5′-CCAGCACTTCACCCATCAGTT-3′	127
*β-actin*	(F): 5′-GTCAGGTCATCACTATCGGCAAT-3′ (R): 5′-AGAGGTCTTTACGGATGTCAACGT-3′	147

**Table 2:** The primer sequences of *TNF-α*, *IL-1β*, *IL-6*, *iNOS*, *COX-2*, and *β-actin* (rat).

Gene	Sequence	Length (bp)
*TNF-α*	(F): 5′-CCACGCTCTTCTGTCTACTG-3′ (R): 5′-GCTACGGGCTTGTCACTC-3′	145
*IL-1β*	(F): 5′-TGTGATGTTCCCATTAGAC-3′ (R): 5′-AATACCACTTGTTGGCTTA-3′	131
*IL-6*	(F): 5′-AGCCACTGCCTTCCCTAC-3′ (R): 5′-TTGCCATTGCACAACTCTT-3′	156
*iNOS*	(F): 5′-CACCCAGAAGAGTTACAGC-3′ (R): 5′-GGAGGGAAGGGAGAATAG-3′	186
*COX-2*	(F): 5′-AGAGTCAGTTAGTGGGTAGT-3′ (R): 5′-CTTGTAGTAGGCTTAAACATAG-3′	170
*β-actin*	(F): 5′-GTCAGGTCATCACTATCGGCAAT-3′ (R): 5′-AGAGGTCTTTACGGATGTCAACGT-3′	147

## Abbreviations

PD	Parkinson’s disease
DA neurons	Dopaminergic neurons
SN	Substantia nigra
TH	Tyrosine hydroxylase
SNpc	Substantia nigra pars compacta
